# Chronic Hepatitis C Therapy in Liver Cirrhosis Complicated by Telaprevir-Induced DRESS

**DOI:** 10.1155/2014/380424

**Published:** 2014-08-20

**Authors:** Omar Y. S. Mousa, Rossa Khalaf, Rhonda L. Shannon, Chukwuma I. Egwim, Scott A. Zela, Victor Ankoma-Sey

**Affiliations:** ^1^State University of New York Upstate Medical University, 750 E Adams Street, Syracuse, NY 13210, USA; ^2^Department of Medicine, Liver Associates of Texas, P.A, 6410 Fannin Street No. 225, Houston, TX 77030, USA; ^3^Department of Pathology, St. Luke's Episcopal Hospital, 6720 Bertner Avenue, Houston, TX 77030, USA; ^4^Gastroenterology/Hepatology, Liver Associates of Texas, P.A, 6410 Fannin Street No. 225, Houston, TX 77030, USA

## Abstract

Drug reaction with eosinophilia and systemic symptoms (DRESS) is a rare yet severe adverse drug-induced reaction with up to 10% mortality rate. Recent clinical trials reported an association between DRESS and telaprevir (TVR), an NS3/4A protease inhibitor of chronic hepatitis C (CHC) virus genotype 1. Its diagnosis is challenging given the variable pattern of cutaneous eruption and the myriad internal organ involvement. We present two patients who are middle-aged, obese, and white with CHC cirrhosis. They both developed a progressive diffuse, painful pruritic maculopapular rash at weeks 8 and 10 of CHC therapy with TVR, Peg-Interferon alfa-2a, and Ribavirin. They had no exposures to other medications that can cause this syndrome. Physical exam and labs and skin biopsy supported a “Definite” clinical diagnosis of DRESS, per RegiSCAR criteria. Thus Telaprevir-based triple therapy was discontinued and both patients experienced rapid resolution of the systemic symptoms with gradual improvement of eosinophilia and the skin eruption. These two cases illustrate the paramount importance of having a high index of suspicion for TVR-induced DRESS, critical for early diagnosis. Immediate discontinuation of TVR is essential in prevention of a potentially life-threatening complication. Risk factors for development of DRESS in patients receiving TVR remain to be elucidated.

## 1. Case Presentations

### 1.1. Patient A

A 65-year-old White lady with a known history of liver cirrhosis secondary to chronic hepatitis C (CHC) infection presented with worsening painful pruritic maculopapular eczematous rash over a period of 2 weeks. The rash started during the eighth week of triple therapy for CHC using telaprevir (TVR), Peg-Interferon alfa-2a, and Ribavirin (P/R). It was progressive, started on the trunk, and spread to the rest of her body over a period of 3 weeks prior to hospitalization. It was associated with mild facial edema, fatigue, nausea, diarrhea, hearing loss the in right ear, weight gain of 15 lbs over a 3 week period, and bilateral lower extremity swelling.

Although the rash was progressive, our patient insisted to complete the 12 weeks of TVR therapy as she had undetectable hepatitis C virus (HCV) RNA levels at weeks 4 and 8. The skin eruption continued to worsen during the 5 days following completion of TVR therapy, despite Ribavirin dose reduction and application of Triamcinolone and Hydrocortisone creams as well as Atarax orally. She also received Procrit for anemia and her hypertension and diabetes mellitus type II were controlled with Lasix, Lisinopril, Hydrochlorothiazide, and Metformin. She has a significant history of anaphylactoid reaction secondary to monosodium glutamate in the past. She has no previous family history of DRESS.

**Figure 1:**
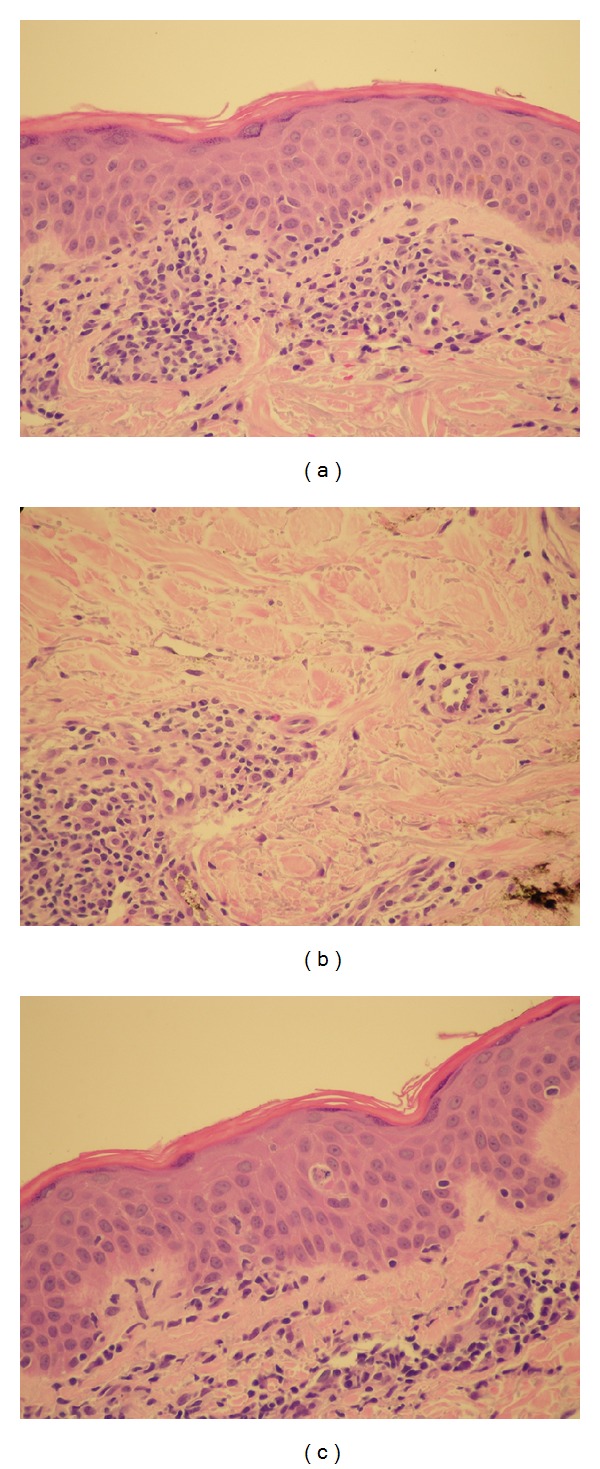


**Figure 2:**
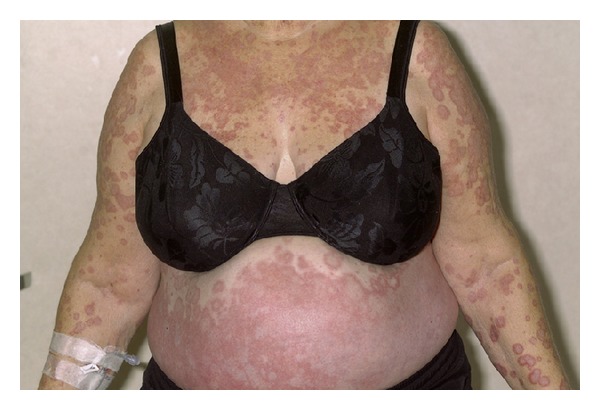
Patient A: extensive diffuse maculopapular rash involving the trunk.

Her physical examination was significant for fever 101.7F and an extensive diffuse maculopapular eczematous rash throughout the body including the head, trunk, extremities, and oral mucous membranes (Figure 2). She also had facial edema, 2+ lower extremities edema, and cervical lymphadenopathy. Exam was otherwise unremarkable. Laboratory tests on admission are shown in Table 1 as patient A. Chest X-ray showed mild left sided atelectasis and blood and urine cultures were negative. Punch biopsy of the skin revealed superficial perivascular dermatitis and focal interface dermatitis. The perivascular inflammation is predominantly lymphocytic and includes rare eosinophils (Figures 1(a), 1(b), and 1(c)).

The CHC triple therapy was discontinued on admission (week 12 of triple therapy) as a severe adverse drug reaction, particularly DRESS was suspected. She received skin care with Triamcinolone and Hydrocortisone creams and medical therapy with Hydroxyzine, magic mouthwash, and Ranitidine. Lasix, Lisinopril, Hydrochlorothiazide, and Metformin were held due to acute kidney injury. She also received empiric antibiotic coverage initially with Cefepime and Vancomycin. Systemic steroids were not used. Our patient experienced rapid resolution of the fever and swelling with gradual improvement of the skin rash. She was discharged home one week later with significant improvement in the rash and the kidney function returning to baseline.

### 1.2. Patient B

Our second patient is a 51-year-old White gentleman with a known history of CHC cirrhosis, portal hypertension, esophageal varices, and diabetes mellitus. He presented to the clinic at week 10 of CHC triple therapy (TVR/P/R) with diffuse pruritic rash over the head, trunk, upper and lower extremities that worsened over a 2-week period. He denied any other symptoms except for subjective fever, anxiety, and depression. He has no previous history of allergic reactions and he was not taking any medications that can be suspected as the cause of a similar rash.

**Figure 3:**
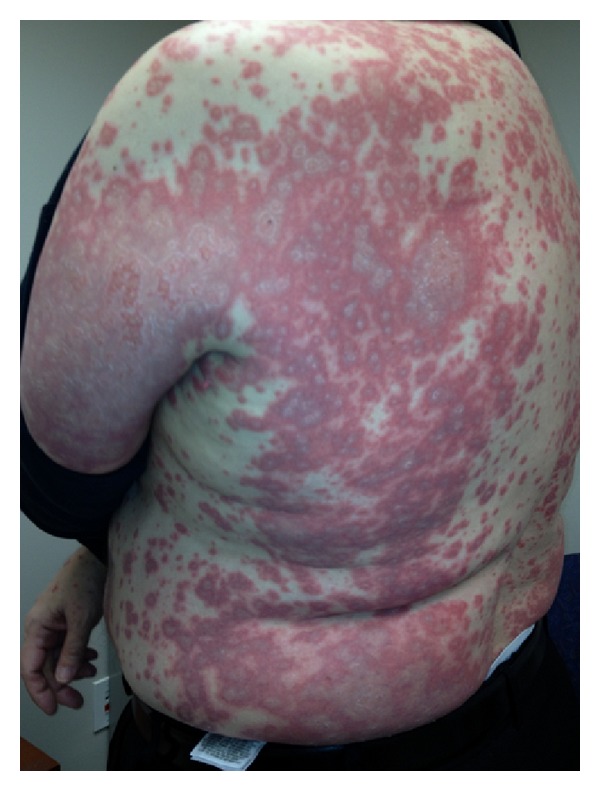
Patient B: diffuse pruritic maculopapular rash involving approximately >80% of the body surface area. Involvement of the trunk is shown in this figure.

On physical examination, he had a diffuse pruritic maculopapular rash involving approximately >80% of the body surface area (Figure 3), occipital lymphadenopathy, facial edema, and shallow oral mucosal ulcers. He was admitted to the hospital and the CHC triple therapy was discontinued immediately as the diagnosis of DRESS versus Steven Johnson syndrome was suspected. Laboratory investigations on admission are shown in Table 1, as patient B. Chest X-ray was clear and blood and urine cultures showed no evidence of infection. The skin biopsy showed similar superficial perivascular dermatitis and interface dermatitis, as well as mild spongiosis and basal cell layer liquefaction. No eosinophils were seen.

However, the patient's hospital stay was complicated by fever 101.4F and acute atrial fibrillation on day five of admission. He was asymptomatic and hemodynamically stable and complete blood count showed eosinophilia 19%. He received Amiodarone and supportive care with IV fluids, Pepcid, and Benadryl in addition to Metformin and Actos for glycemic control. The patient refused topical steroid therapy and continued Nadolol for portal hypertension. His skin rash improved significantly and the other symptoms resolved during his hospital stay. He was discharged home 10 days after a close follow-up.

## 2. Discussion

Chronic hepatitis C virus (CHC) infection is an asymptomatic yet serious disease with a challenging era of antiviral therapies. In the year 2011, two direct acting agents (DAA), telaprevir (TVR) and boceprevir, which are NS3-4A protease inhibitors, were approved by the FDA to change the landscape of hepatitis C therapy. Even though the sustained virologic responses were significantly improved as shown in phases 2 and 3 of clinical trials [1–3], still serious adverse events, drug resistance, and nonresponse to treatment remain as challenging obstacles.

Earlier we presented two cases of drug rash with eosinophilia and systemic symptoms (DRESS), a severe adverse drug-induced reaction. This syndrome developed in two of our patients during triple therapy for CHC with TVR, Peg-Interferon alfa-2a, and Ribavirin (P/R). Although DRESS is rare, with an estimated incidence from 1 : 1000 to 1 : 10,000 [4], it is serious with up to 10% mortality rate [5]. Previous reports from clinical trials showed that TVR can be associated with this potentially life-threatening disease [6]. DRESS is a novel term introduced in the year 1996, prior to which various names were used to describe the same clinical presentation as a “hypersensitivity syndrome.” Different medications contributed to the causality of this syndrome, which added to the confusion in its pathophysiology [7]. For example, this syndrome was previously described as anticonvulsant hypersensitivity syndrome given the predominance of anticonvulsant medications in literature review as a cause of this syndrome [7]. However, DRESS is a preferred term as recent and future antiviral medications could be potential causes as well.

Both patients had a similar presentation, with the diffuse pruritic rapidly progressing skin rash flaring at week 8 of TVR therapy. This presentation occurred at a later time than previous reports that stated a usual presentation at 2–6 weeks [4, 6]. The skin eruption was accompanied by fever, eosinophilia, lymphadenopathy, facial edema, and hepatic or renal involvement, with persistence of the skin rash and delayed resolution despite the withdrawal of TVR. All these factors are significantly associated with probable/definite cases of DRESS according to the RegiSCAR scoring system (European registry of severe cutaneous adverse reaction) [4]. Despite the fact, they are not predictive of the severity of the disease.

The diagnosis of such skin rash is challenging due to the variable patterns of cutaneous eruption and the myriad of internal organ involvement, besides the overlap in presentation with other categories of drug-induced skin reactions. A pathognomonic pattern of this rash is not provided in the literature yet [4]. Diagnostic procedures like the skin biopsy showed nonspecific findings. Exclusion of other categories in the differential diagnosis that can mimic this presentation was possible with negative laboratory results for antinuclear antibodies (ANA), Mycoplasma/Chlamydia, and cultures of the blood and urine.

We graded both cases as life-threatening systemic reaction with reference to data from phases 2 and 3 of TVR clinical trials, as both DRESS and Steven Johnson syndrome (SJS) were suspected [5, 8]. However, further clinical review of the cases and deeper study of the rash shifted the diagnosis against SJS, as less than one site of mucous membranes was involved with no blistering or epidermal detachment. In addition, we applied two scoring systems, RegiSCAR and the Naranjo adverse drug reaction probability scale, that were developed to help establish a diagnosis of such life-threatening drug reaction by grading the DRESS cases as “No,” “Possible,” “Probable,” or “Definite” cases [4, 9, 10]. Both systems showed different results that still supported the diagnosis of DRESS in our patients. They were classified as “Definite” cases of DRESS using the RegiSCAR criteria (patient A score of 8 versus patient B score of 8, where “score > 5” is considered as a “Definite” case). However, they were found to be “Probable” cases of DRESS using the Naranjo probability scale (patient A score of 6 versus patient B score of 7, where “score of 5–8” is considered as a “Probable” case). Refer to Tables 2 and 3 for detailed application of the RegiSCAR scoring system and the Naranjo probability scale for both case presentations [9–11].

Management of DRESS is particularly based on the immediate withdrawal of the suspected offending medication, telaprevir in our cases. This is accompanied by hospital admission for close follow-up and supportive therapy with hydration, skin care with topical steroids, and systemic antihistamines [5, 8]. This management approach helped resolve all the symptoms during the hospitalization of both patients, with return of the liver and kidney function to baseline within a week of hospital discharge. In addition, both patients achieved early virologic responses below level of detection at week 12 (EVR12LOD) and maintained undetectable HCV RNA levels thereafter, despite the discontinuation of CHC triple therapy between the weeks 8 and 12. We enforce the significance of early detection and permanent discontinuation of TVR and P/R for the treatment of this serious reaction. We did not consider intravenous immunoglobulins for the management of DRESS due to lack of evidence-based consensus on this treatment in addition to previously reported adverse outcomes [12].

Of noteworthy is that our two patients had liver cirrhosis, which can make them less tolerant to the CHC triple therapy with TVR compared to noncirrhotics, and thus may become susceptible to some life-threatening complications. In addition both patients were obese with a BMI of 32.9 and 35.4 kg/m^2^. Literature review including the regiSCAR study lacks specific data regarding a relation between the development of DRESS and obesity in CHC cirrhotic patients. There is no evidence of any predictive factors that increase the risk of such serious complications. We suggest that obesity and liver cirrhosis might be risk factors for the development of DRESS during TVR-based CHC triple therapy. Other risk factors remain to be elucidated.

To conclude, it is essential to have a high index of suspicion among healthcare professionals for the diagnosis of DRESS in patients receiving chronic hepatitis C therapy. Early diagnosis and prompt management is paramount in improving the outcome, in a potentially life-threatening complication.

## Figures and Tables

**Table 1:** Laboratory tests during the hospital admission for both patients A and B.

Laboratory tests	Day of admission		Day 3 of admission		Day 9 of admission		Discharge day	
Patient	A	B	A	B	A	B	A	B
Hemoglobin (g/dL)	10.7	12.9	8.9	11.9	8.7	13.9	10.2	10.7
Platelet count (×10^3^/mm^3^)	69	48	75	46	129	67	121	64
Eosinophils (%)	15	11	20	12	18	28	21	22
White blood cell count (×10^3^/mm^3^)	4.9	2	6.3	2.4	7.5	5.1	6.7	3.3
Serum creatinine mg/dL)	1.67	0.63	1.18	0.59	0.81	0.54	1.27	0.49
AST (U/L)	39	86	35	86	37	38	57	54
ALT (U/L)	30	48	26	56	27	29	33	31
Total bilirubin (mg/dL)	1.3	2.5	0.9	1.6	0.8	2.2	1.0	1.1
Albumin (g/dL)	2.9	3.3	2.3	2.9	2.4	2.7	2.7	2.3
Alkaline phosphatase	93	59	98	46	143	57	95	62

**Table 2:** Naranjo adverse drug reaction probability scale for each DRESS case. Total scores in cases A and B were 7 and 6, respectively. Scores from 5 to 8 suggest Probable cases of DRESS. Bold cells are positive findings [11].

Number	The Naranjo adverse drug reaction probability scale	Patient A			Patient B		
		Yes	No	Do not know	Yes	No	Do not know
1	Are there previous conclusive reports of this reaction?	**+1**	0	0	**+1**	0	0
2	Did the adverse event appear after the drug was given?	**+2**	−1	0	**+2**	−1	0
3	Did the adverse reaction improve when the drug was discontinued or a specific antagonist was given?	**+1**	0	0	**+1**	0	0
4	Did the adverse reaction reappear upon readministering the drug?	+2	−1	**0**	+2	−1	**0**
5	Were there other possible causes for the reaction?	−1	**+2**	0	−1	**+2**	0
6	Did the adverse reaction reappear upon administration of placebo?	−1	+1	**0**	−1	+1	**0**
7	Was the drug detected in the blood or other fluids in toxic concentrations?	+1	0	**0**	+1	0	**0**
8	Was the reaction worsened upon increasing the dose? Or, was the reaction lessened upon decreasing the dose?	+1	0	**0**	+1	0	**0**
9	Did the patient have a similar reaction to the drug or a related agent in the past?	+1	**0**	0	+1	**0**	0
10	Was the adverse event confirmed by any other objective evidence?	**+1**	0	0	+1	**0**	0
	**Total**	**+7**			**+6**		

**Table 3:** RegiSCAR scoring system for DRESS classification. Final scores 2-3 = Possible, 4-5 = Probable, and >5 = Definite case of DRESS [9, 10]. Bold cells are positive findings.

Score	Patient A				Patient B			
	−1	0	1	2	−1	0	1	2
Fever ≥38.5°C	No/U	**Yes**	—	—	No/U	**Yes**	—	—
Enlarged lymph nodes	—	No/U	**Yes**	—	—	No/U	**Yes**	—
Eosinophilia		No/U				No/U		
Eosinophils	—	—	0.7–1.49 × 10^9^ L^−1^	>1.5 × 10^9^ L^−1^	—	—	**0.7–1.49 × **10^9^ ** ** **L** ^−1^	>1.5 × 10^9^ L^−1^
Eosinophils, if leukocytes < 4.0 × 10^9^ L^−1^	—	—	10–19.9%	**≥20%**	—	—	10–19.9%	**≥20%**
Atypical lymphocytes	—	No/U	**Yes**	—	—	**No/U**	—	—
Skin involvement:								
Skin rash extent (% body surface area)	—	No/U	**>50%**	—	—	No/U	**>50%**	—
Skin rash suggesting DRESS	No	U	**Yes**	—	No	U	**Yes**	—
Biopsy suggesting DRESS	No	**Yes/U**	—	—	No	Yes/U	—	—
Organ involvement:								
Liver	—	No/U	**Yes**	—	—	No/U	**Yes**	—
Kidney	—	No/U	**Yes**	—	—	**No/U**	Yes	—
Muscle/heart	—	**No/U**	Yes	—	—	**No/U**	Yes	—
Pancreas	—	**No/U**	Yes	—	—	**No/U**	Yes	—
Other organ	—	**No/U**	Yes	—	—	**No/U**	Yes	—
Resolution ≥15 days	No/U	**Yes**	—	—	No/U	**Yes**	—	—
Evaluation of other potential causes:								
Antinuclear antibody	—	—	—	—	—	—		—
Blood culture								
Serology for HAV/HBV/HCV								
Chlamydia/mycoplasma								
If none positive and ≥3 of above negative			Yes				**Yes**	
**Total Score**	**8**				**8**			
